# An elevational shift facilitated the Mesoamerican diversification of Azure‐hooded Jays (*Cyanolyca cucullata*) during the Great American Biotic Interchange

**DOI:** 10.1002/ece3.10411

**Published:** 2023-08-15

**Authors:** John E. McCormack, Molly M. Hill, Devon A. DeRaad, Eliza J. Kirsch, Kelsey R. Reckling, Marquette J. Mutchler, Brenda R. Ramirez, Russell M. L. Campbell, Jessie F. Salter, Alana K. Pizarro, Whitney L. E. Tsai, Elisa Bonaccorso

**Affiliations:** ^1^ Moore Laboratory of Zoology Occidental College Los Angeles California USA; ^2^ Biodiversity Institute and Department of Ecology and Evolutionary Biology University of Kansas Lawrence Kansas USA; ^3^ Ornithology Department Natural History Museum of Los Angeles County Los Angeles California USA; ^4^ Laboratorio de Biología Evolutiva, Colegio de Ciencias Biológicas y Ambientales Universidad San Francisco de Quito Quito Ecuador

**Keywords:** biogeography, birds, integrative taxonomy, ornithology, phylogeography, systematics

## Abstract

The Great American Biotic Interchange (GABI) was a key biogeographic event in the history of the Americas. The rising of the Panamanian land bridge ended the isolation of South America and ushered in a period of dispersal, mass extinction, and new community assemblages, which sparked competition, adaptation, and speciation. Diversification across many bird groups, and the elevational zonation of others, ties back to events triggered by the GABI. But the exact timing of these events is still being revealed, with recent studies suggesting a much earlier time window for faunal exchange, perhaps as early as 20 million years ago (Mya). Using a time‐calibrated phylogenetic tree, we show that the jay genus *Cyanolyca* is emblematic of bird dispersal trends, with an early, pre‐land bridge dispersal from Mesoamerica to South America 6.3–7.3 Mya, followed by a back‐colonization of *C. cucullata* to Mesoamerica 2.3–4.8 Mya, likely after the land bridge was complete. As *Cyanolyca* species came into contact in Mesoamerica, they avoided competition due to a prior shift to lower elevation in the ancestor of *C. cucullata*. This shift allowed *C. cucullata* to integrate itself into the Mesoamerican highland avifauna, which our time‐calibrated phylogeny suggests was already populated by higher‐elevation, congeneric dwarf‐jays (*C. argentigula*, *C. pumilo*, *C. mirabilis*, and *C. nanus*). The outcome of these events and fortuitous elevational zonation was that *C. cucullata* could continue colonizing new highland areas farther north during the Pleistocene. Resultingly, four *C. cucullata* lineages became isolated in allopatric, highland regions from Panama to Mexico, diverging in genetics, morphology, plumage, and vocalizations. At least two of these lineages are best described as species (*C. mitrata* and *C. cucullata*). Continued study will further document the influence of the GABI and help clarify how dispersal and vicariance shaped modern‐day species assemblages in the Americas.

## Graphical abstract

*Cyanolyca* jays are representative of general trends in avian movements during the Great American Biotic Interchange: dispersal from Mesoamerica to South America during a pre‐land bridge pulse before 5 million years ago, followed by dispersal from South America to Mesoamerica after the land linkage. Upon back‐colonizing Mesoamerica, the Azure‐hooded Jay (*C. cucullata*) diversified on a Pleistocene landscape already sculpted by geologic processes and populated by higher‐elevation congeners.

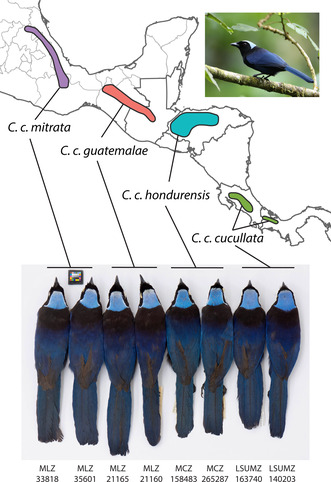

## 1 INTRODUCTION

The highlands of Mexico and Central America (i.e., Mesoamerican highlands) are a biodiversity hotspot (Myers et al., 2000). The mechanisms underlying the generation of biodiversity in this region have been the subject of numerous investigations (Bryson et al., 2011; DeRaad, Applewhite, et al., 2023; Mastretta‐Yanes et al., 2015; Ornelas et al., 2010; Sullivan et al., 2000; Venkatraman et al., 2019; Zarza et al., 2016). While vicariance seems to have played a major role in speciation—whether via mountain uplift (Bryson Jr et al., 2012; Bryson Jr & Riddle, 2012; Castoe et al., 2009) or Pleistocene glacial cycles (Gutiérrez‐Rodríguez et al., 2022)—dispersal into and out of Mesoamerica has also been a key factor structuring communities (López‐García & Morrone, 2022; Ornelas et al., 2014).

The Great American Biotic Interchange (GABI)—the mixing of flora and fauna between North and South America that occurred after uplift of the Panamanian land bridge (Stehli & Webb, 2013; Weir et al., 2009)—was perhaps the most extraordinary dispersal event in the history of the Americas. The GABI profoundly affected local and regional species composition, niche partitioning, natural selection pressures, extinction, and speciation. Fossils (Webb, 1991) and, later, molecular phylogenies (Cody et al., 2010; Weir et al., 2009) document dispersal events between continents triggered by the GABI, but the exact timing of faunal exchange has been debated. Much work has focused on the time period between 3 and 4 million years ago (Mya; Coates et al., 1992; O'Dea et al., 2016), but other studies suggest prior connections throughout the last 20 My (Bacon et al., 2015; Montes et al., 2015; Weir et al., 2009). Individual case studies are still adding to our knowledge of when and how the GABI took place (Barker, 2007; Cortés‐Ortiz et al., 2003; Navarro‐Sigüenza et al., 2020; Núñez‐Zapata et al., 2016; Rull, 2011; Sedano & Burns, 2010).

The jay genus *Cyanolyca* presents a compelling system for investigating the role of the GABI in dispersal, vicariance, and species accumulation. The genus consists of nine species spread across the Neotropics. Nested within an otherwise South American group, one species, the Azure‐hooded Jay (*C. cucullata*), appears to have back‐colonized Mesoamerica (Bonaccorso, 2009; Bonaccorso & Peterson, 2007). The implied dispersal order may conform to general findings that an early, pre‐land bridge pulse of bird dispersal occurred mostly from North America to South America (Cadena et al., 2007; Weir et al., 2009), whereas a more recent pulse, after the rise of the land bridge, occurred mostly in the opposite direction from South America to North America (Bonaccorso & Guayasamin, 2013; Cadena et al., 2007; Weir et al., 2009). Despite these compelling patterns, the *Cyanolyca* phylogeny has never been time‐calibrated, and divergences among allopatric lineages of *C. cucullata* in Mesoamerica have not been addressed with DNA data, so it is not clear whether the biogeography lines up with these identified pulses of dispersal.

**FIGURE 1:**
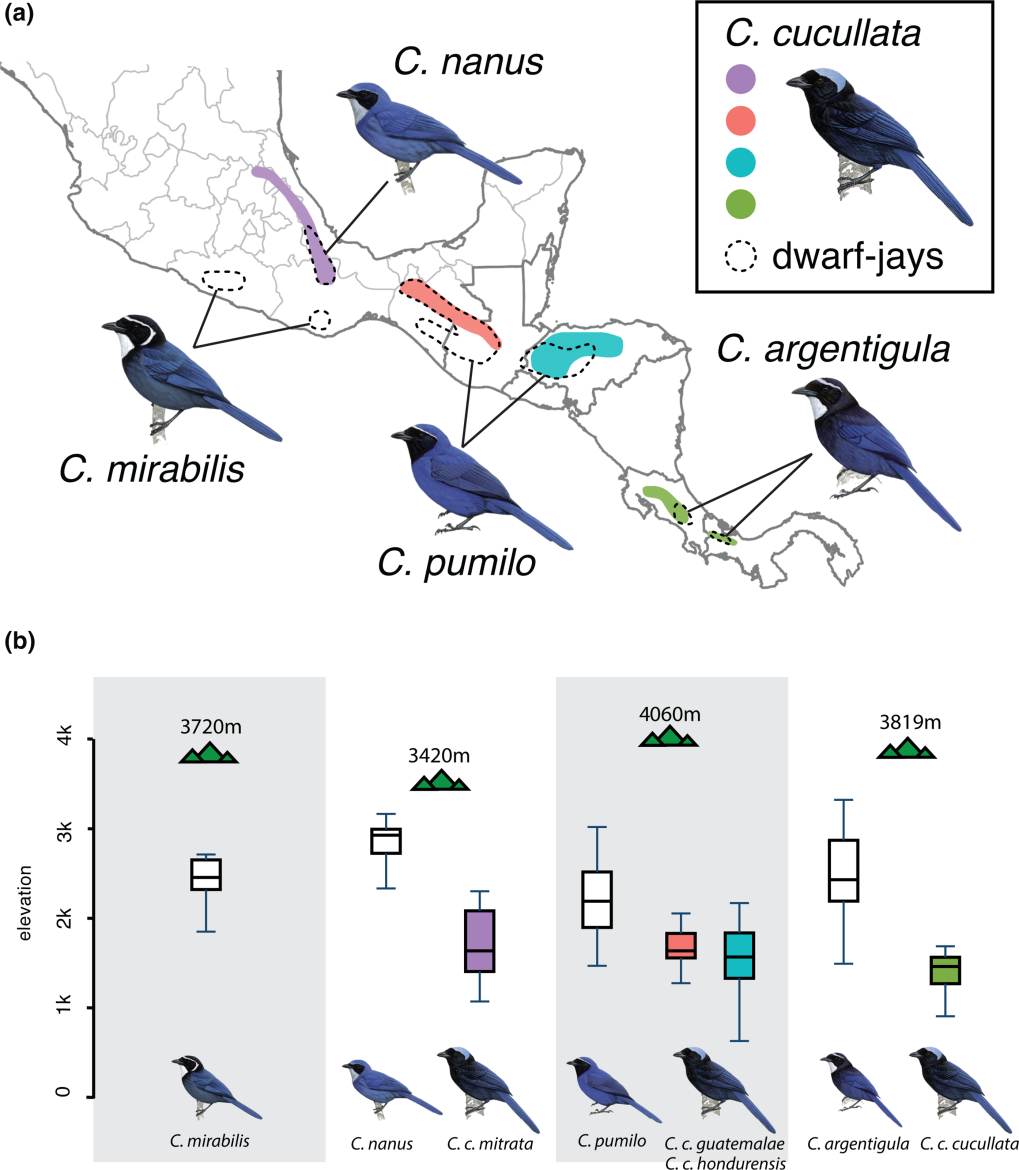
(a) Range overlap between *Cyanolyca cucullata* and the four *Cyanolyca* dwarf‐jay species in Mesoamerica. (b) Elevational ranges of co‐occurring species. Mountain icon indicates highest peak. Bird illustrations used with permission from Birds of the World Handbook.

The back‐colonization of *C. cucullata*, in particular, also presents an opportunity to investigate the consequences of the GABI on species accumulation in Mesoamerica via elevational zonation, a key feature of community assembly in the Neotropical highlands. Studies have focused on the role of interspecific competition in driving elevational zonation (Diamond, 1970; Terborgh & Weske, 1975), but an alternate hypothesis (called species sorting or stochastic sorting; Cadena, 2007; Freeman, 2015) states that zonation may simply result from secondary contact between species with pre‐existing elevational differences. In the case of *Cyanolyca*, *C. cucullata* now inhabits disjunct highland forests across Mesoamerica in close proximity to four congeneric species (that we collectively call dwarf‐jays because of their smaller size), which never left Mesoamerica (Figure 1). *C. cucullata* inhabits lower elevations than the dwarf‐jays (Bonaccorso, 2009), but it is not clear whether this represents an elevational shift that preceded secondary contact, which would support the species sorting prediction, or whether current elevational ranges were attained closer to the time of contact between species, which would support interspecific competition.

**FIGURE 2:**
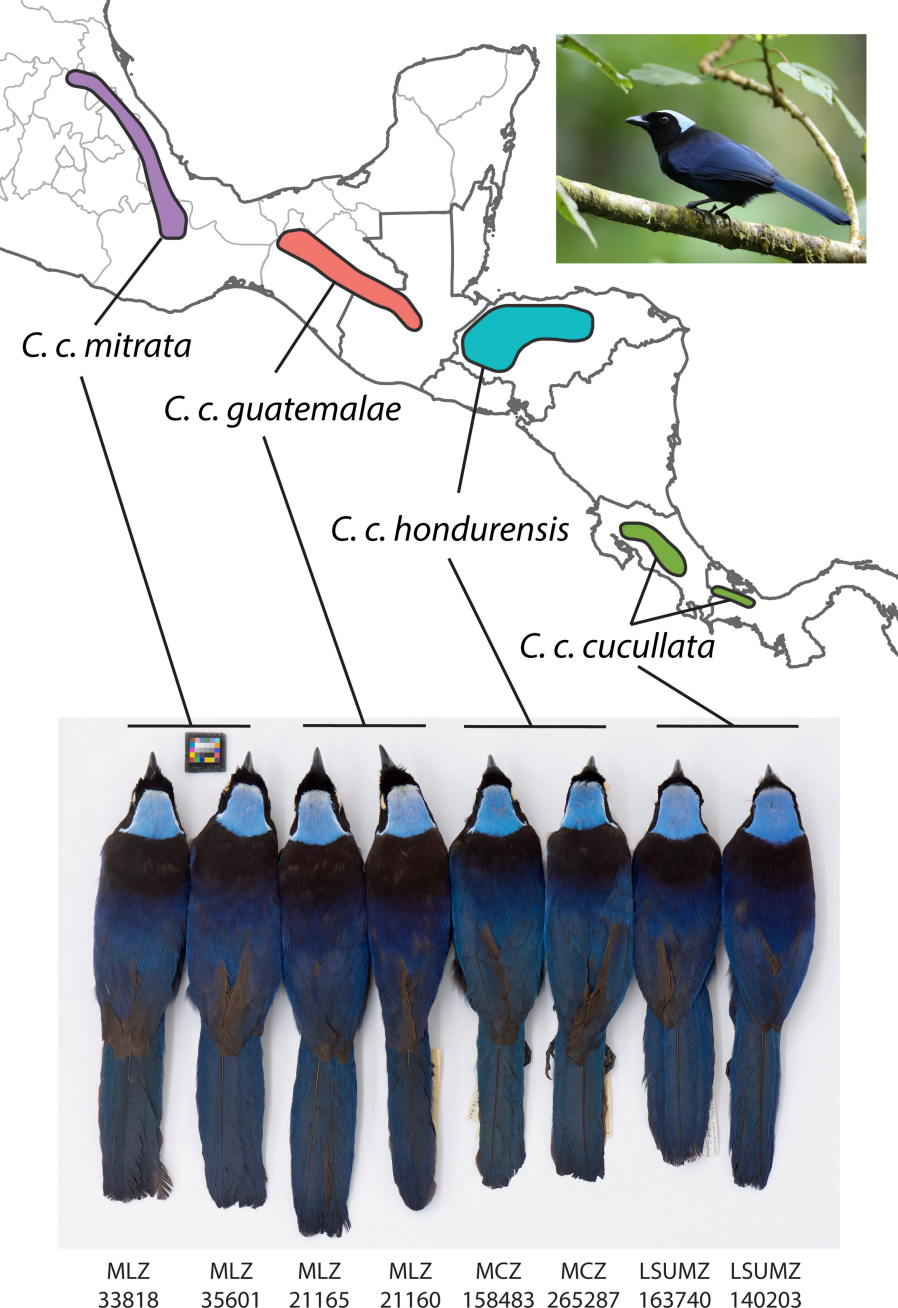
Geographic range of *C. cucullata* with subspecies and representative specimens for each lineage. For museum codes, see Table S1. Live bird photo: *C. c. cucullata*, Monteverde, Costa Rica ML204340451, Hal and Kirsten Snyder.

To investigate the role of the GABI on dispersal, divergence, and elevational zonation in *Cyanolyca*, we estimated a time‐calibrated phylogeny for the genus. First, we tested whether dispersal events between continents line up with previously identified pulses of dispersal resulting from the GABI. Next, to understand how the back‐colonization of Mesoamerica by *C. cucullata* impacted elevational zonation among *Cyanolyca* species, we combined the phylogeny with elevation data to test whether *C. cucullata* shifted to lower elevation prior to its arrival to Mesoamerica (supporting species sorting) or later, after encountering congeners (supporting interspecific competition). Finally, we conducted an integrative taxonomic study of the four allopatric *C. cucullata* lineages distributed throughout Mesoamerica (Figure 2) to assess their biogeographic history and the potential for unrecognized species‐level diversity within this polytypic species.

## 2 MATERIALS AND METHODS

### 2.1 Taxonomy and sampling

We carried out species‐level genetic sampling of the genus *Cyanolyca* (Table S1) using tissue samples or toe pads from each of the nine recognized species (Clements et al., 2022): *C. argentigula*, *C. pumilo*, *C. mirabilis*, *C. nanus*, *C. cucullata*, *C. armillata*, *C. pulchra*, *C. turcosa*, and *C. viridicyanus*. For *C. cucullata*, we additionally sampled from four distinct highland areas in Mesoamerica (Figure 2): the nominate *C. c. cucullata* in Costa Rica and Panama, *C. c. hondurensis* in Honduras, *C. c. guatemalae* in Guatemala and Chiapas, Mexico, and *C. c. mitrata* in Oaxaca and Hidalgo, Mexico. To assess phenotypic variation within *C. cucullata*, we analyzed a large set of museum specimens and vocalizations (see File S1).

### 2.2 Phylogenetics and time calibration

We assembled a mitochondrial DNA (mtDNA) dataset to build a phylogeny of *Cyanolyca* and added nuclear genomic data to create a phylogeny of *C. cucullata*. Our goal was not to obtain a complete phylogeographic assessment of *Cyanolyca* with dense geographic sampling, but rather to assess divergence times among species and to define distinctive lineages in *C. cucullata* to provide a framework for assessing phenotypic variation.

For the nuclear genomic data, we followed the protocol for target enrichment of ultraconserved elements (UCEs) outlined in Faircloth et al. (2012). Briefly, we obtained DNA from tissues or toe pads from each *C. cucullata* subspecies and an outgroup, the Beautiful Jay (*C. pulchra*). For degraded toepad samples, DNA was extracted using a phenol–chloroform protocol (Tsai et al., 2020) in a clean room, while frozen tissue samples were extracted using a DNeasy spin‐column kit (Qiagen). DNA from tissue was sonicated to 400–600 base pairs (bp). We then performed library preparation using Kapa HyperPrep library preparation kits (F. Hoffman‐La Roche Ltd), affixing unique iTru sequence tags (Glenn et al., 2019) to each individual. Samples were pooled and enriched for UCEs using the myBaits UCE Tetrapods 5Kv1 bait kit from Daicel Arbor Biosciences, followed by an 18‐cycle recovery PCR and sequencing on an Illumina HiSeq.

After sequencing, samples were demultiplexed and the reads were cleaned using illumiprocessor (Faircloth, 2013), a wrapper for trimmomatic v.0.39.1 (Bolger et al., 2014). Cleaned UCE data were then mapped to a high‐quality reference genome assembly for the closely related California Scrub‐Jay (*Aphelocoma californica*; DeRaad, Escalona, et al., 2023, in press) using Bowtie2 v.2.3.3.1 (Langmead & Salzberg, 2012). We used the Genome Analysis Tool Kit (GATK) v.3.8.1 (McKenna et al., 2010) to identify variable sites based on homologous mapping of UCE reads across the reference genome. We filtered single nucleotide polymorphisms (SNPs) using GATK and VCFtools v0.1.15 (Danecek et al., 2011) and removed SNPs with mean depth < 5 or >200, a quality score divided by allele depth (QD) < 2, strand bias (FS) > 40, mapping quality (MQ) < 20, or HaplotypeScore (the number of haplotypes containing the SNP across all samples) > 12. We required SNPs to be biallelic and thinned the dataset to a minimum distance of 1000 bp between SNPs. Using the R packages SNPfiltR v1.0.0 (DeRaad, 2022) and vcfR v1.12.0 (Knaus & Grünwald, 2017), we removed invariant SNPs (generated when all samples were homozygous alternate compared with the reference genome) and set a 100% completeness threshold, retaining a final dataset of 1075 unlinked SNPs shared across all samples with zero missing genotypes.

We converted this SNP dataset to a binary nexus file using the R package ape v5.5 (Paradis & Schliep, 2019), which we used as input to reconstruct a species tree using SNAPP implemented via BEAST2 v2.7.1 (Bouckaert et al., 2014). We ran two identical SNAPP replicates, each with an MCMC length of 5M generations, sampling from the posterior every 1K generations. For each run, we visually confirmed convergence and effective sample size >200 for all estimated parameters using Tracer (Rambaut et al., 2018). We discarded the first 1M generations as burn‐in, concatenated the 4000 postburn‐in trees from each chain into a single treefile using LogCombiner (distributed as part of BEAST2), and visualized all species trees sampled from the posterior distribution using DensiTree (Bouckaert, 2010).

For mtDNA, we skimmed reads from the off‐target UCE sequences (Raposo do Amaral et al., 2015) and assembled *cyt b* sequences for all *Cyanolyca* species and *ND2* for *C. cucullata* following Salter et al. (2020). Briefly, we used MITObim v1.9 (Hahn et al., 2013), a PERL wrapper around MIRA 4.0.2 (Chevreux et al., 1999), to reconstruct mtDNA genomes for our samples using a Florida Scrub‐Jay (*Aphelocoma coerulescens*) mtDNA genome from NCBI Genbank as a reference (NC_05146.1). We used Geneious Prime (https://www.geneious.com) to annotate mtDNA sequences by aligning them to the reference and extracted the *ND2* and *cyt b* genes for all samples. Using *ND2* allowed us to combine our data with existing *ND2* data for *C. cucullata* publicly available on Genbank (Table S1). We created a time‐calibrated Bayesian phylogeny for *cyt b* with BEAST, using a relaxed clock and a molecular substitution rate of 0.0105 substitutions per site per lineage to convert genetic divergence to time, based on a rate estimated from fossils and well‐timed biogeographic events (Weir & Schluter, 2008). For *ND2*, we generated a Bayesian tree with posterior probabilities in MrBayes 3.2.7 (Ronquist et al., 2012), using the HKY substitution model and runs of 200,000 generations, sampling every 10 generations with a 10% burn‐in. Convergence was assessed with Tracer.

### 2.3 Elevation data

We downloaded locality information for *Cyanolyca* species from eBird (2021) and manually curated occurrence records to remove obviously aberrant records. Using the R program *raster* (Hijmans et al., 2013), we spatially thinned these data to retain only one observation per square km and to remove the top and bottom 5% of the distribution of elevational records. We extracted elevation data for the remaining 5617 records. For each species, we visualized the mean and 25%–75% range of the elevational distributions using box plots. While observational records can be geographically biased, our results accord with descriptive accounts of elevation ranges from the literature (Billerman et al., 2020) and are likely to capture the kind of broad differences in elevational zonation we address in this study.

### 2.4 Morphology

We measured 56 adult *C. cucullata* specimens housed in natural history collections (see File S1): eight *C. c. mitrata*, 18 *C. c. guatemalae*, 10 *C. c. hondurensis*, and 20 *C. c. cucullata*. We measured unflattened wing cord to the nearest 0.5 mm using a wing ruler, and tail, tarsus, bill length (front of the nares to the tip of the mandible), bill width, and bill depth (at the front of nares) to the nearest 0.1 mm with digital calipers. We also took measurements from 126 adult birds of four dwarf‐jay species (*C. pumilo*, *C. mirabilis*, *C. argentigula*, and *C. nanus*) to compare intraspecific differentiation within *C. cucullata* to interspecific variation between co‐occurring congeners. For statistical analysis, we checked for normality and then calculated differences in univariate traits and reduced the dimensionality of the data with principal components analysis (PCA) of the correlation matrix, visualizing major axes of variance (PCs) in a two‐way scatterplot in Stata Intercooled v14. We tested for significance with Student's *t*‐tests.

### 2.5 Vocalizations

We obtained 52 recordings of two common call types (see File S1), using nine recordings from xeno‐canto (https://xeno‐canto.org/), six from the Florida Museum of Natural History, and 37 from the Macaulay Library, representing 13 individuals from *C. c. mitrata*, 10 from *C. c. guatemalae*, and 29 from *C. c. cucullata*. As is typical for corvids, *C. cucullata* has a number of call types and variants. The Handbook of Birds of the World (Fitzgerald, 2020) describes one of the main call types as *djhenk*, with a geographic variation called *oink*. In all the available recordings of these birds, the *djhenk* call was only noted in *C. c. cucullata*, whereas only the *oink* call was noted in birds from the three allopatric subspecies to the north. Based on this evidence, we analyzed the *djhenk* and *oink* calls under the assumption that they are homologous call types. We excluded poor recordings and recordings with overlapping calls from multiple individuals. We quantified the calls using RavenPro software v1.6.3 (Cornell Lab of Ornithology), measuring peak frequency (in Hz, the frequency at the point of maximum volume), peak power density (in dB FS), average power density (dB FS), aggregate entropy (in bits, quantifying the disorder of the call), and length (in frames). Calls are typically given in bursts of a few repetitions with no pauses. For each recording, we averaged every repetition of the call within one burst. We analyzed this variation using a PCA, visualizing PC1 as a box plot and assessing significance with a two‐tailed Student's *t*‐test in Stata Intercooled v14.

### 2.6 Plumage color

We measured plumage color from 29 museum specimens representing the four allopatric *C. cucullata* subspecies. On each bird, we took measurements on the blue part of the back and the forecrown using a JAZ spectrophotometer (Ocean Optics), taking three measurements for each patch to obtain an average. Spectral curves were averaged and smoothed with the R package *pavo* (Maia et al., 2019). Standard brightness, hue, and chroma were quantified and compared among lineages with box plots. To generate a descriptive summary of plumage evolution within *C. cucullata*, we plotted putatively derived plumage features on the phylogeny with reference to the outgroup *C. pulchra*, assuming parsimony.

## 3 RESULTS

### 3.1 Phylogeny and elevation

**FIGURE 3:**
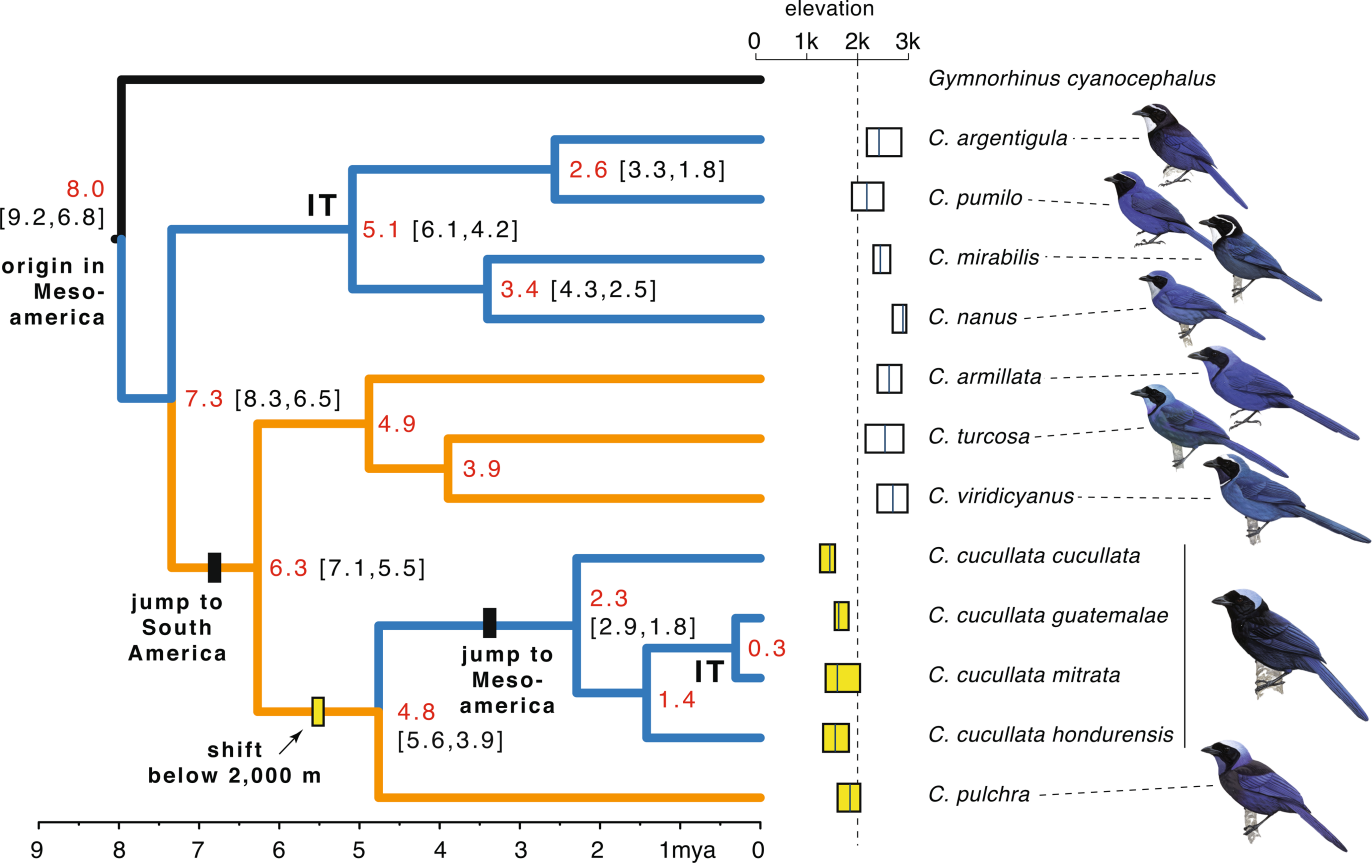
Time‐calibrated *cyt b* phylogeny of *Cyanolyca* showing estimated divergence times in millions of years (red text) and 95% highest probability density (in brackets) as well as splits across the Isthmus of Tehuantepec (IT) and elevational ranges (box plots show mean in meters and 25%–75% range; yellow boxes = mean < 2000 m). Posterior probability (pp) for all nodes ≥.98. Blue lines = Mesoamerican; orange lines = South American. Bird illustrations used with permission from Birds of the World Handbook.

**FIGURE 4:**
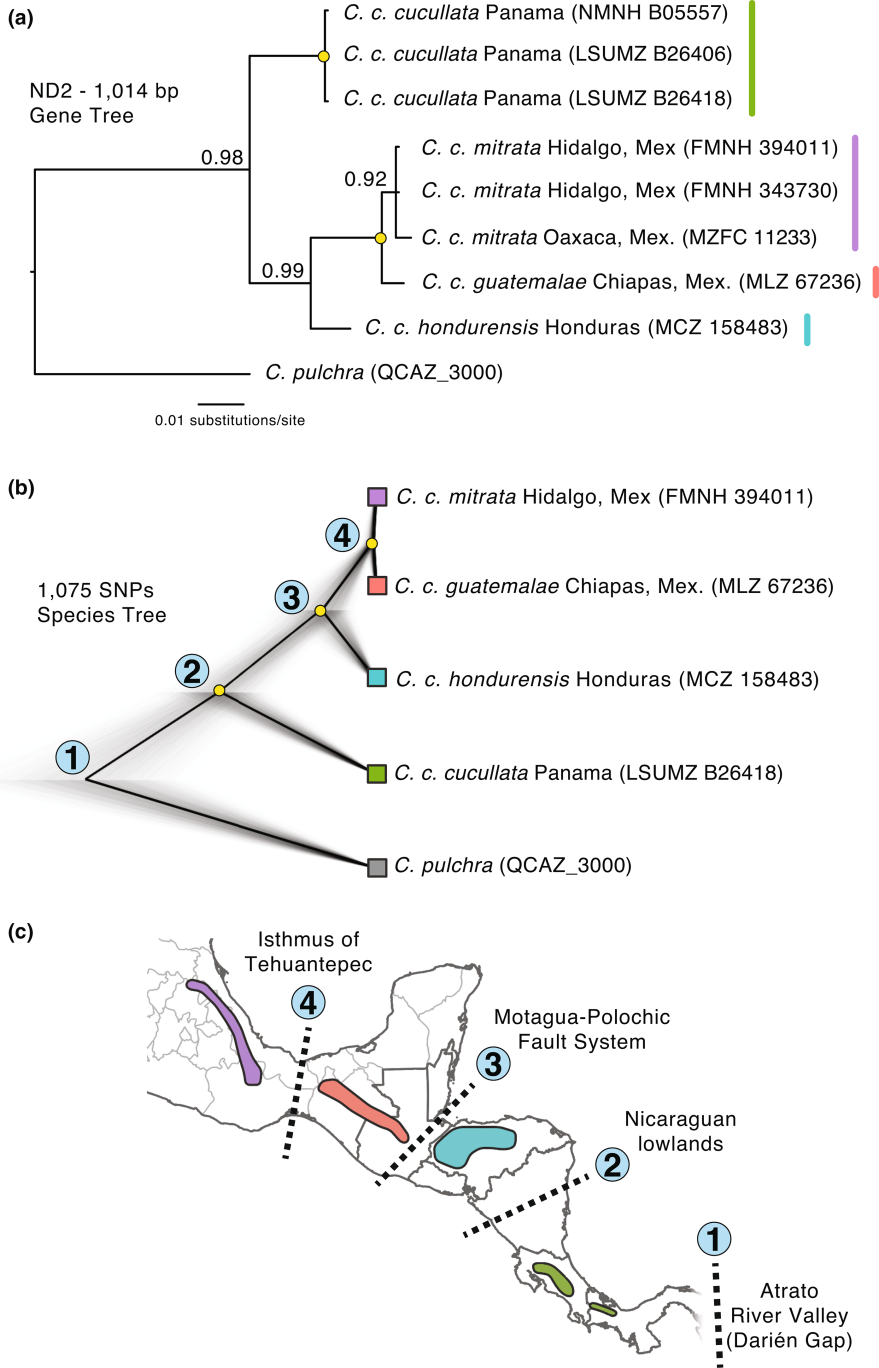
Phylogeography of *C. cucullata*. Yellow dots are nodes with pp = 1.0. (a) Bayesian *ND2* tree; (b) Species tree from 1075 SNPs drawn from UCE data; and (c) the order of biogeographic splits relating to the node labels in panel (b).

The time‐calibrated *Cyanolyca* mtDNA tree for the *cyt b* gene (Figure 3) based on 1143 bp was well‐resolved (posterior probabilities ≥ .98) and suggests that the stem lineage leading to all modern *Cyanolyca* lineages arose around 8 Mya (95% highest probability density [95HPD] = 6.8–9.2 Mya). Because a prior study placed the origin of *Cyanolyca* in Mesoamerica (Fernando et al., 2017), we can infer a dispersal event into South America 6.3–7.3 Mya (95HPD = 5.5–8.3 Ma), with a subsequent recolonization of Mesoamerica by *C. cucullata* 2.3–4.8 Mya (95HPD = 1.8–5.6 Ma). Assessing elevational distributions with respect to the phylogeny revealed that only *C. cucullata* and *C. pulchra* have mean elevations below 2000 m. When two *Cyanolyca* lineages coinhabit the same mountain range in Mesoamerica, *C. cucullata* always inhabits significantly lower elevation than the co‐occurring dwarf‐jay species (Figure 1b). For *C. cucullata*, the SNAPP tree based on 1075 SNPs and a better‐sampled mtDNA tree based on 1014 bp of the *ND2* gene (Figure 4) agree in topology, showing deep divergence between a lineage in Panama and Costa Rica (*C. c. cucullata*) and a clade including three lineages to the north (*C. c. hondurensis* + *C. c. guatemalae* + *C. c. mitrata*).

### 3.2 Morphology

**FIGURE 5:**
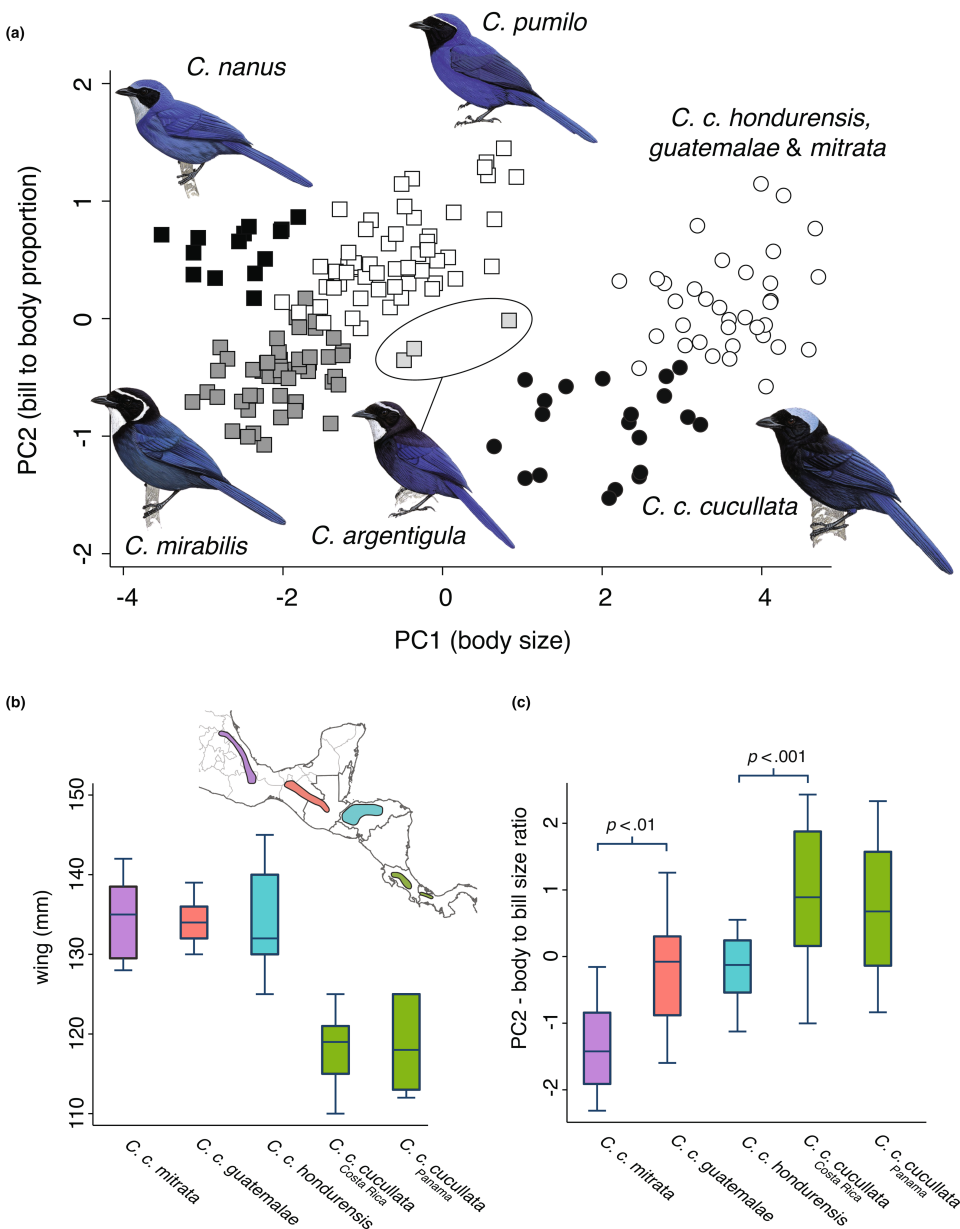
(a) Morphological PCA of *C. cucullata*, plus the four dwarf‐jay species. Differences in (b) wing length and (c) bill‐to‐body ratio among *C. cucullata* lineages. Illustrations reproduced with permission from Birds of the World Handbook.

The morphology PCA identified two major axes of variation explaining 94% of the overall morphological variation (PC1 = 87%; PC2 = 7%). Based on the loadings of individual variables on the PC axes (Table S2), PC1 reflects overall body size, while PC2 is associated with bill‐to‐body proportion. A scatterplot of PC1 and PC2 shows separation between *C. c. cucullata* and all northern *C. cucullata* lineages (Figure 5a). Although males averaged larger than females (PC1 by sex: *p* < .001, *t* = 4.52, df = 48), patterns of variation were similar between sexes (Figure S1), so our results combine data from both sexes. Overall, *C. c. cucullata* is smaller than northern birds, with a proportionally larger bill (Figure 5a). Wing length for *C. c. cucullata* shows no overlap with northern birds (Figure 5b). *C. c. mitrata* has a significantly smaller bill in relation to its body size compared with its nearest neighbor across the Isthmus of Tehuantepec, *C. c. guatemalae* (Figure 5c; *t*‐test for morphological PC2: *p* < .01, *t* = 2.89, df = 23), and raw bill measurements of *C. c. mitrata* were also significantly smaller for bill width (mean *C. c. mitrata* = 9.7 mm, mean *C. c. guatemalae* = 10.3 mm, *p* = .04, *t* = −2.22, df = 24) and close to significant for bill length (mean *C. c. mitrata* = 18.9 mm, mean *C. c. guatemalae* = 19.6 mm, *p* < .06, *t* = −1.96, df = 24) and depth (mean *C. c. mitrata* = 11.0 mm, mean *C. c. guatemalae* = 11.4 mm, *p* < .08, *t* = −1.80, df = 24).

### 3.3 Vocalizations

**FIGURE 6:**
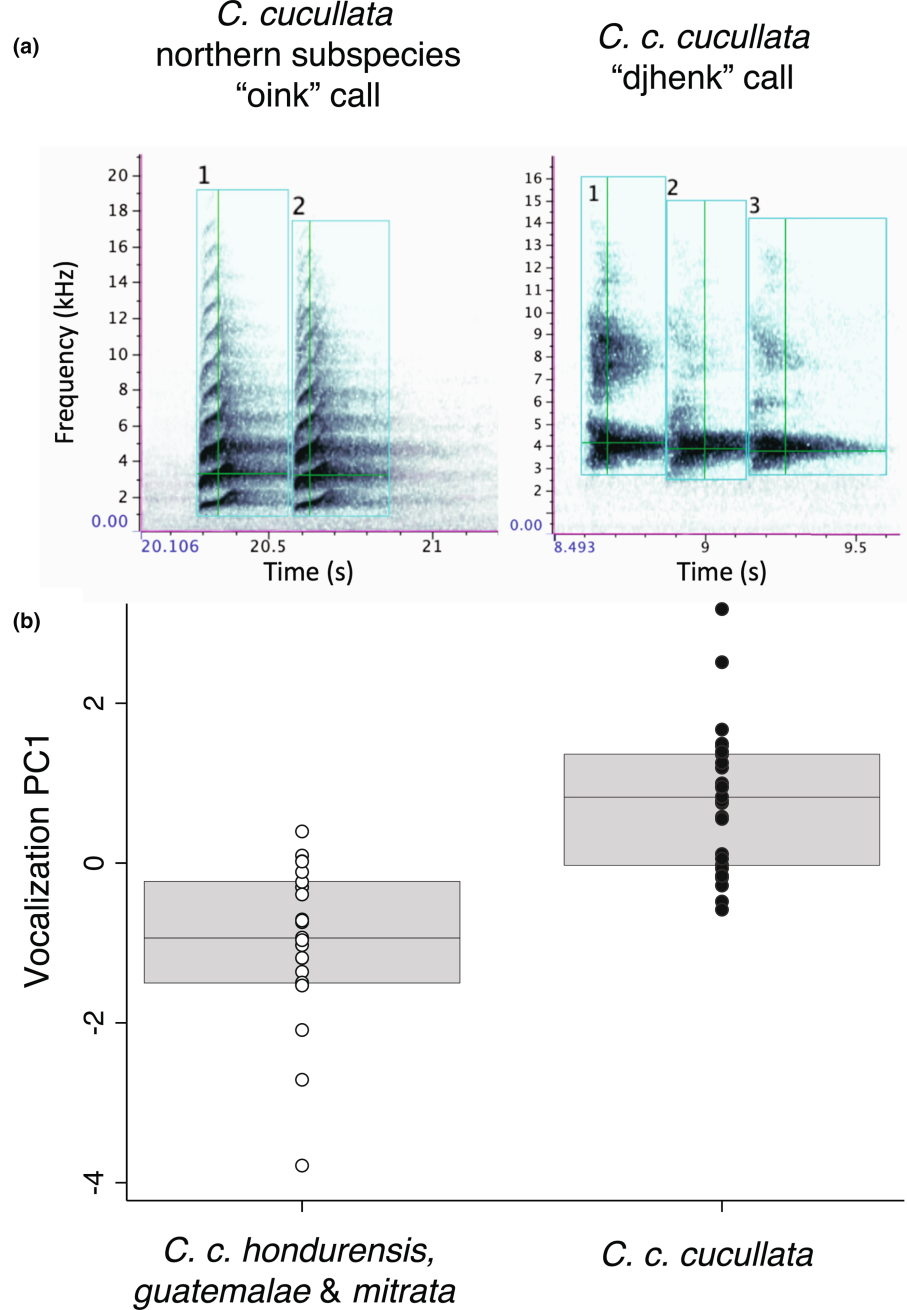
Comparison of the *djhenk* and *oink* call types. (a) On left, spectrogram of ML362557151, an example of an *oink* call of *C. c. mitrata*, generated by RavenPro software. On right, spectrogram of ML398519931, an example of *djhenk* call of *C. c. cucullata*. The blue boxes show the portion of the calls used to take measurements, while the green crosses mark the location of the peak power density (i.e., the intersection between peak frequency and peak time). (b) Box plot of PC1 values for both call types. Means differed significantly (*t*‐test: *t* = −6.81; *p* < .001).

Average power density and peak power density were highly correlated (0.94), so we dropped peak power density from our analysis. A PCA produced two axes with eigenvalues >1 explaining 67% of the overall variation (Table S3). PC1 associated positively with average power density and call length and negatively with spectral disorder. PC2 associated positively with peak frequency and negatively with average power density. Overall, the *djhenk* calls of *C. c. cucullata* were higher frequency and more concentrated in specific frequencies, whereas *oink* calls of northern populations showed harmonics spread over a wider, but lower frequency (Figure 6). PC1 values differed significantly between *C. c. cucullata* and the combined northern lineages (*t*‐test: *t* = −6.81; *p* < .001, df = 50). The *oink* calls of the three northern *C. cucullata* subspecies showed no significant pairwise comparisons among them.

### 3.4 Plumage color

**FIGURE 7:**
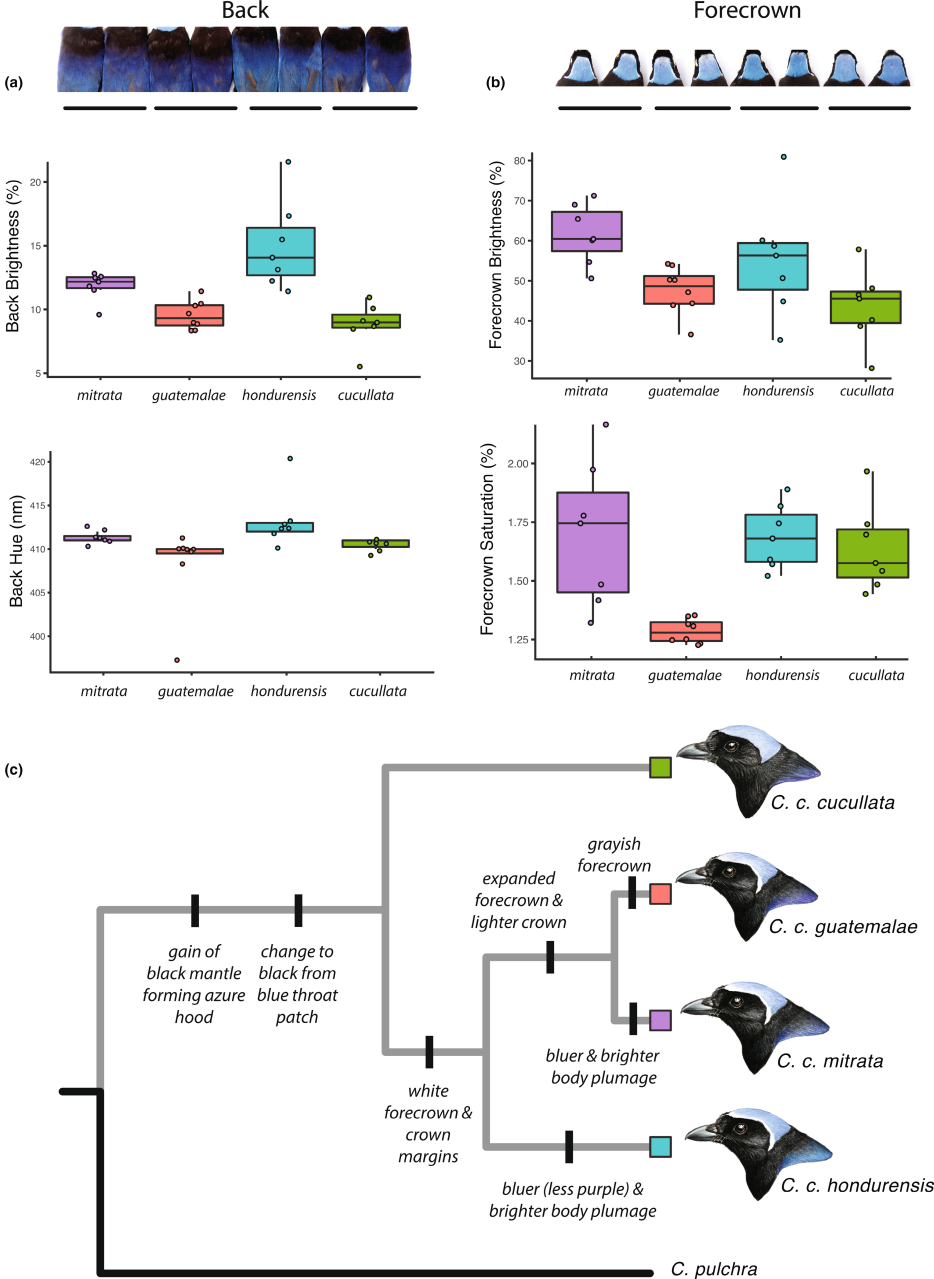
Plumage differences among *C. cucullata* lineages. (a) back and (b) forecrown, and (c) implied changes in plumage traits along the phylogeny. Representative illustrations for each lineage by Marky Mutchler.

Back brightness and hue, and forecrown brightness and saturation showed differences among some lineages (Figure 7). *C. c. hondurensis* had the brightest body plumage (Figure 7a), which was obvious in specimens. Differences in *C. c. mitrata* body color were more subtle, but overall *C. c. mitrata* also had a brighter and bluer color than *C. c. guatemalae* and *C. c. cucullata*, which appeared darker. While the northern lineages *C. c. mitrata* and *C. c. guatemalae* both had an expanded light forecrown, the forecrown of *C. c. guatemalae* had little saturation and appeared grayer (Figure 7b).

## 4 DISCUSSION

The Great American Biotic Interchange radically reshaped the biota of the Americas. The biogeographic history of *Cyanolyca* shows how dispersal during the GABI led to both diversification and species accumulation. Prior to the final uplift of the Panamanian land bridge, *Cyanolyca* appears to have first colonized South America 6.3–7.3 Mya, after which several species diversified in the proto‐Andes that were forming in northwestern South America. One of these lineages, the ancestor to *C. pulchra* and *C. cucullata*, became isolated and shifted to lower elevation below 2000 m. This elevation shift, combined with the final linkage of the Americas ca. 3–4 Mya (Coates et al., 1992), permitted the stem ancestor of all *C. cucullata* lineages to disperse over the lowlands at the border of Panama and Colombia (the Darién Gap) to the mountains of Panama and Costa Rica. Then, over the last ca. 2 My, *C. cucullata* made successive jumps northward over lowland barriers to inhabit allopatric mountain ranges from Honduras to central Mexico. In doing so, *C. cucullata* came into contact with other *Cyanolyca* species (the dwarf‐jays) that had never left Mesoamerica. The same shift to lower elevation that may have facilitated *C. cucullata*'s dispersal to Mesoamerica apparently also allowed for elevational zonation and co‐existence.

### 4.1 Deep history of *Cyanolyca*: implications for the Great American Biotic Interchange

Many studies of the GABI have focused on a time period 2.5–4 Mya for the final linkage of North and South America via the uplift of the Panamanian land bridge (O'Dea et al., 2016). However, recent geological (Montes et al., 2015) and comparative molecular work (Bacon et al., 2015; Pinto‐Sánchez et al., 2012; Weir et al., 2009) suggest that this linkage might not have been sudden and that dispersal instead went through pulses over the last 20 My. In the case of *Cyanolyca*, a prior study pointed to a Mesoamerican origin for the genus (Fernando et al., 2017). Our phylogeny, therefore, estimates the timing of the dispersal of a new *Cyanolyca* lineage from Mesoamerica to South America perhaps as old as 6.3–7.3 Mya, an event which left behind a lineage in Mesoamerica that would later diversity into the modern dwarf‐jays. This time period overlaps the early burst of bird dispersal from North to South America between 7 and 10 Mya previously identified by Weir et al. (2009) across several bird families. The timing of this event speaks to the nature of the connection between North and South America prior to the land bridge. *Cyanolyca* jays, being neither habitat generalists nor frequent long‐distance fliers, are not among the top candidates for making long, overwater journeys. While it is impossible to rule out a rare, long‐distance dispersal event, our results lend support to the hypothesis of a proto‐land bridge or a shallow island chain connecting the continents at this time.

In whatever manner this ancient stem lineage to the South American *Cyanolyca* jays made its way south, it would have found suitable forest habitat in the proto‐Andes, which had been rising over the course of the prior 50 My (Hoorn et al., 2010; Pérez‐Escobar et al., 2022). Later, ca. 5 Mya, the ancestor to *C. pulchra* and *C. cucullata* likely became isolated on the northwestern coastal slope of the proto‐Andes. The lower elevational ranges of both *C. pulchra* and *C. cucullata* compared with other *Cyanolyca* suggest that this ancestor made the shift to lower elevation habitat prior to *C. cucullata* recolonizing Mesoamerica. As suggested previously by Bonaccorso (2009), such an elevational shift might have provided the very means for dispersal across the Panamanian lowlands into the mountains of Panama and Costa Rica, where the evolutionary story of *C. cucullata* in Mesoamerica begins.

### 4.2 The Pleistocene journey of *C. cucullata* northward through Mesoamerica

Studies of the origin of biodiversity in the Mesoamerican highlands have rightly focused on in situ vicariance and speciation via barrier formation (Bryson Jr & Riddle, 2012; Caviedes‐Solis & Leaché, 2018; Marshall & Liebherr, 2000; McCormack et al., 2008), but dispersal into this region has also been important to species accumulation (López‐García & Morrone, 2022; Ornelas et al., 2014). For *C. cucullata*, our phylogeny suggests a history of differentiation in the highlands of Mesoamerica as this lineage was making successive jumps north across a landscape already sculpted by geologic processes (Barrier et al., 1998; Ferrari et al., 1999; Rogers et al., 2002). The first of these jumps (Figure 4c), ca. 2.3 Mya, was over the Nicaraguan lowlands to the highlands of Honduras, perhaps facilitated by a cooler Pleistocene climate that expanded highland forest to lower elevation, decreasing the distance between forest patches. The next jump, at ca. 1.4 Mya, was across the Motagua–Polochic Fault System that separates the highlands of Honduras and Guatemala, an important barrier for many organisms (Castoe et al., 2009; Rovito et al., 2012).

The most recent and final jump was across the Isthmus of Tehuantepec ca. 300,000 years ago, perhaps during the penultimate (Illinoian) glaciation. Using a mitochondrial mutation rate similar to the one we assumed for this study, a previous comparison of bird divergences across the Isthmus (Barber & Klicka, 2010) found two main pulses of divergence, one quite recent ca. 0.13 Mya (0–0.34 Mya confidence interval), which overlaps the 95HPD of the Isthmus split in *C. cucullata*, and an older pulse at 1.6 Mya (0.7–3.2 Mya). Even older dates have been estimated for cross‐Isthmus divergence, from 3 to 4 Mya (Tsai et al., 2019; Venkatraman et al., 2019) to as old at 5 Mya (Ornelas et al., 2010). The exact age of the Isthmus is not certain, but geologic evidence suggests an origin via tectonic plate movement in the late Miocene ca. 6 Mya (Barrier et al., 1998), potentially followed by marine incursions as recently as 2–3 Mya at the Pliocene–Pleistocene transition (Mulcahy et al., 2006; Sullivan et al., 2000). While the split of *C. cucullata* across the Isthmus is much more recent, clearly reflecting dispersal across a barrier long since formed, there is a much older cross‐Isthmus split reflected in the dwarf‐jay group of *Cyanolyca*, dating to ca. 5 Mya (Figure 3). Assuming the ranges of these species have not shifted much since their diversification, this deeper split might relate to the origin of the Isthmus and further illustrates the long history of habitation of the dwarf‐jay species in this region compared with *C. cucullata*.

A significant cohort of species that currently inhabits the Mexican Transition Zone (Halffter, 1976), a broadly defined area lying between the Nearctic and Neotropical Zones that includes both lowlands and highlands, can trace their origins to northward dispersal events triggered by the GABI (López‐García & Morrone, 2022). *C. cucullata* was part of this cohort and, for a highland humid forest specialist, among the few that achieved maximal northern penetration into the Sierra Madre Oriental. Some arid, open‐habitat bird species, like the Inca Dove (*Columbina inca*) managed to spread all the way into the United States after the GABI (Sweet & Johnson, 2015). But other lowland forest specialists—such as the Great Curassow (*Crax rubra*; Hosner et al., 2016), the Northern Barred Woodcreeper (*Dendrocolaptes sheffleri*; Navarro‐Sigüenza et al., 2020), and the Gray‐chested Dove (*Leptotila cassinii*; Johnson & Weckstein, 2011)—only reached the southern coastal lowlands of Mexico. Similarly, several humid forest tanager species moved north after the GABI, but many did not make it past Nicaragua, with only a few reaching as far north as southern Mexico (Burns & Racicot, 2009; Sedano & Burns, 2010). Some highland forest generalists like the White‐throated Thrush (*Turdus assimilis*) colonized the northern Mexican highlands (Núñez‐Zapata et al., 2016). The Gray‐barred Wren (*Campylorhynchus megalopterus*), a highland species of pine‐oak forests, also back‐colonized North America and reached as far north as the Trans‐Mexican Volcanic Belt (Barker, 2007). As effective as the northern dispersal of *C. cucullata* has been for a highland humid forest species, there are still seemingly suitable areas that it did not manage to colonize, like the Sierra Madre del Sur in Guerrero, Mexico, although extinction cannot be ruled out as an explanation for this absence.

### 4.3 A shift to lower elevation preceded elevational zonation in Mesoamerican *Cyanolyca*

As *C. cucullata* entered Central America and moved north, it encountered a landscape that was already populated by congenerics, the dwarf‐jays—the descendent species of the original lineage left behind after the colonization of South America. Modern co‐existence between *C. cucullata* and these four species now appears to be mediated through elevational zonation, but how did this arise? Elevational zonation of birds in the Neotropics has long been explained through interspecies competition (Diamond, 1970; Terborgh & Weske, 1975), but an alternate explanation is that the elevational zonation we observe today is the result of community assembly by species that were already complementary in their elevational distributions before they came into contact (Rice & Pfennig, 2007). This latter explanation, which forms a sort of null hypothesis for elevational zonation, has been referred to as species sorting or stochastic sorting and appears to explain some cases of elevational zonation, like in the *Buarremon* brush‐finches of the Neotropical highlands (Cadena, 2007). Yet, a recent, multispecies study uncovered strong evidence for interspecific competition driving elevational divergence in the Neotropics (Freeman, 2015).

In *Cyanolyca*, it appears that a shift to lower elevation habitat below 2000 m in the ancestor of *C. cucullata* and *C. pulchra* preceded elevational zonation between *C. cucullata* and the co‐occurring dwarf‐jay species in Mesoamerica, supporting the species sorting hypothesis. The implied shift to lower elevation habitat prior to the dispersal of *C. cucullata* across Mesoamerica is based on a parsimonious reading of the phylogeny, where *C. cucullata* and its sister species *C. pulchra* are unique in the genus for inhabiting mean elevations below 2000 m. Although our mtDNA phylogeny was based on only one sample per species, prior work including nuclear introns also supports the same topology (Bonaccorso, 2009). Nevertheless, it is not impossible to imagine that *C. cucullata* and *C. pulchra* independently made shifts to lower elevation more recently, so it is also worth considering the history of the elevational ranges in the dwarf‐jays.

All extant dwarf‐jays (*C. nanus*, *C. pumilo*, *C. mirabilis*, and *C. argentigula*) occupy comparatively high elevations. Especially telling is *C. mirabilis*, which does *not* co‐occur with *C. cucullata*. If interspecific competition were driving elevation differences, we might expect *C. mirabilis* to have a lower or wider elevational range, but instead its range is similarly high and narrow like other dwarf‐jays, again supporting a pre‐existing elevational preference. Given that the extant species of dwarf‐jays arose prior to 2 Mya, they likely inhabited their current high‐elevation niche before the arrival of *C. cucullata* to Mesoamerica. This interpretation rests on several assumptions, including general niche conservatism through evolutionary time, and ignores the possibility of extinction. Still, until more information becomes available, perhaps from data on species' ecologies and interactions, the weight of the evidence in *Cyanolyca* points toward species sorting, and not interspecific competition, driving elevational zonation and permitting species co‐existence. While interspecific competition has undoubtedly played a large role in elevational zonation in many cases, *Cyanolyca* presents a counterexample, similar to the *Buarremon* brush‐finches (Cadena, 2007), where pre‐existing, complementary elevation ranges have allowed for the co‐existence of closely related species after secondary contact (also see Reijenga et al., 2023).

### 4.4 Integrative taxonomy and species limits in *C. cucullata*

Our genetic sampling for *C. cucullata* included few individuals, preventing a formal approach to genomic species delimitation. Nevertheless, we found support for deep and concordant divergences within *C. cucullata* in both mtDNA and the genomic species tree. While the allopatric ranges of these lineages make it impossible to test for reproductive isolation, they also mean that the lineages have little opportunity for ongoing gene flow, which strengthens the case for reciprocal monophyly despite our limited sampling. It is especially unlikely that further sampling would erode the monophyly of three of the lineages, which occur on long phylogenetic branches: (1) *C. c. cucullata*; (2) *C. c. hondurensis*, and (3) *C. c. guatemalae* + *C. c. mitrata*.

As for phenotypic evolution, as *C. cucullata* was colonizing new mountain ranges over the last 2 My, it was also diverging in morphology, plumage, and vocalizations. Morphologically, the nominate *C. c. cucullata* has little overlap with other subspecies in body size and shape, as well as individual measurements like wing length. This level of morphological divergence is on par with differences among the four currently recognized *Cyanolyca* dwarf‐jay species that are distributed over a similar area (Figure 5a). We also confirmed that *C. c. cucullata* gives a unique regional call variant. *C. cucullata* has acquired multiple derived plumage traits, including a black mantle and black throat, which combine to create the distinctive hooded, or bonneted, appearance (Figure 7c). From there, white crown margins developed in the ancestor of the three northern lineages. Later still, the white forecrown expanded in the ancestor of *C. c. guatemalae* and *C. c. mitrata*, becoming light gray in *C. c. guatemalae*. The directionality of change is less clear in body color and brightness, but at least two changes have occurred in brightness of the blue color. Only the nominate, *C. c. cucullata*, lacks an implied plumage apomorphy, but this still leaves it diagnosable from other lineages in plumage alone, not to mention morphology and vocalizations.

Taken together, each of the four *C. cucullata* lineages is distinct in genetics and multiple phenotypic traits. Plumage traits seem to be a leading indicator of divergence, in keeping with a comparative study of sister taxa that found that sexual traits evolve at steadier rates compared with ecological traits (Freeman et al., 2023). Current subspecies limits capture the overall variation within this species quite well, with the question being whether the variation is better described as two or more species. Deciding where to draw the line for species status will always be controversial, with many cases lying in the speciation “gray zone” (De Queiroz, 2007). Still, the combination of nonoverlapping morphological variation, discrete vocalization types, and diagnostic plumage, combined with ca. 2 My of genetic isolation, makes a strong case for at least two species within this complex, as described below:

#### 4.4.1 *Cyanolyca mitrata* (Ridgway, 1899)

Type. The type specimen for *C. mitrata* examined by Lesson (1839) was in the collection of Dr. Grégoire Abeillé (1798–1848) of Bordeaux, France. This collection, which was apparently sold after Abeillé's death, has proven untraceable for more than a century (Richmond, 1908). The type location was listed by Lesson as Mexico and was further restricted by Pitelka (1951) to the mountains near Xalapa, Veracruz, based on historical collecting activity and travel routes. Lesson's Latin description (“Corporis parte superiori atterrima [sic]; speculo albo azureoque sub vertice et collo. Dorso, abdomine, alis et cauda azureis.” = Upper part of the body very dark: blue and azure mirror under the crown of head and neck; back, abdomen, wings, and tail blue) does not diagnose *C. mitrata* from *C. cucullata* or clearly distinguish either from the sympatric Dwarf Jay (*C. nanus*). To stabilize the taxonomic identity of *C. mitrata* (Ridgway, 1899) and clarify a type locality, we designate a neotype: an adult male (MLZ:Bird:53628) in the Moore Laboratory of Zoology, Occidental College, Los Angeles, California, collected 2 miles northeast of Huatusco, Veracruz, Mexico, on March 29, 1952, by Chester C. Lamb (Figure S2). This neotype satisfies the requirements for neotype designation in the Code (ICZN, 1999) by clarifying the taxonomic application of the name (Art. 75.3.1), describing, illustrating, and referencing the defining characters of *C. mitrata* and its neotype (Art. 75.3.2), providing data sufficient to ensure recognition of the neotype specimen (Art. 75.3.3), providing grounds for believing that all original type material has been lost and is untraceable (Art. 75.3.4), showing that traits of the neotype are included in the original description (Art. 75.3.5), choosing a neotype collected on the breeding grounds close to the likely original type location (Art. 75.3.6), and depositing the neotype in a recognized scientific institution (Art. 75.3.7).

Diagnosis. Crestless jay with mostly black head and throat except for azure crown becoming whiter toward forecrown, black upper breast and mantle fading into blue body, with blue wings and tail. Compared with *C. cucullata*, *C. mitrata* is larger in all dimensions (25%–75% range of wing length: *C. mitrata* = 125–145 mm; *C. cucullata* = 110–125 mm), has white edges along the azure crown, and gives an *oink* call, compared with the *djhenk* call of *C. cucullata*. Polytypic. The nominate, *C. m. mitrata*, occurs in the highlands of central Mexico west of the Isthmus of Tehuantepec in the states of Oaxaca, Veracruz, and Hidalgo. Subspecies *C. m. guatemalae* (Pitelka, 1951) inhabits the highlands of Chiapas, Mexico, and Guatemala east of the Isthmus of Tehuantepec and has, compared with *C. m. mitrata*, darker and more purplish plumage, a smaller bill on average (25%–75% range of bill length: *C. m. guatemalae* = 17.4–20.3 mm; *C. m. mitrata* = 18.2–20.7 mm), and a more extensive, light‐gray forecrown (cf. white forecrown in *C. m. mitrata*). The southernmost subspecies, *C. m. hondurensis* (Pitelka, 1951), is confined to the highlands of Honduras and has brighter, more blue‐green plumage than *C. m. mitrata*, and a reduced and more demarcated white forecrown. Suggested common name: Azure‐bonneted Jay or Bonneted Jay.

#### 4.4.2 Cyanolyca cucullata (Ridgway, 1885)

Type. USNM 101845 in the National Museum of Natural History, Washington DC. The type locality is Navarro, Costa Rica. Compared with *C. mitrata*, *C. cucullata* is smaller (see above), lacks white margins bordering the azure crown, and gives a *djhenk* call (cf. the *oink* call of *C. mitrata*). Monotypic, although divergence between disjunct populations in Panama and Costa Rica has not been investigated. Suggested common name: retain Azure‐hooded Jay or change to Hooded Jay.

As with other recent cases where widespread jay species have been split into species with smaller geographic ranges (DeRaad et al., 2022; McCormack et al., 2008; Venkatraman et al., 2019), the resulting split would lead to a range‐restricted species of conservation interest. The total range of *C. cucullata*, which inhabits two portions of the Talamanca Range in Costa Rica and Panama, is maybe only ~10,000 km^2^. The Talamanca Range is a center of endemism in Mesoamerica (Marshall & Liebherr, 2000), whose origin as a series of volcanic islands initiated the events leading to the formation of the land bridge connecting the Americas (Coates & Obando, 1996). While most endemic birds inhabiting the Talamanca Range are designated species of least conservation concern by the IUCN, and much of the habitat lies in protected areas, species' ranges are predicted to contract considerably with climate change (Liu et al., 2020), as “the escalator to extinction” (Freeman et al., 2018) pushes suitable habitats upslope and shrinks their size. Continued study of the biogeography of Mesoamerican bird species will help identify evolutionarily significant lineages and clarify the centers of endemism at higher conservation risk.

## AUTHOR CONTRIBUTIONS

**John E. McCormack:** Conceptualization (lead); data curation (supporting); formal analysis (supporting); writing – original draft (lead); writing – review and editing (lead). **Molly M. Hill:** Conceptualization (supporting); formal analysis (supporting); investigation (supporting); writing – original draft (supporting); writing – review and editing (supporting). **Devon A. DeRaad:** Formal analysis (supporting); writing – review and editing (supporting). **Eliza J. Kirsch:** Formal analysis (supporting); writing – review and editing (supporting). **Kelsey R. Reckling:** Data curation (supporting); formal analysis (supporting); writing – review and editing (supporting). **Marquette J. Mutchler:** Data curation (supporting); formal analysis (supporting); writing – review and editing (supporting). **Brenda R. Ramirez:** Data curation (supporting); writing – review and editing (supporting). **Russell M. L. Campbell:** Data curation (supporting). **Jessie F. Salter:** Data curation (supporting); formal analysis (supporting); writing – review and editing (supporting). **Alana K. Pizarro:** Formal analysis (supporting); writing – review and editing (supporting). **Whitney L. E. Tsai:** Data curation (supporting); writing – review and editing (supporting). **Elisa Bonaccorso:** Conceptualization (supporting); data curation (supporting); writing – review and editing (supporting).

## CONFLICT OF INTEREST STATEMENT

The authors declare no conflict of interest.

## Supporting information


File S1:

Appendix S1:

## Data Availability

The raw data underlying the phenotypic analysis are available as an online supplementary file. New mtDNA sequences are deposited in Genbank (OR355544‐OR355559). Raw UCE data are deposited in the SRA (https://www.ncbi.nlm.nih.gov/bioproject/PRJNA996972) and associated code for SNP calling and species‐tree analysis appears on GitHub (https://github.com/DevonDeRaad/cyanolyca.snapp).
