# Supplementary material for: An elevational shift facilitated the Mesoamerican diversification of Azure‐hooded Jays (Cyanolyca cucullata) during the Great American Biotic Interchange
Source: Ecol Evol. 2023 Aug 15;13(8):e10411. doi: 10.1002/ece3.10411 (PMC10425738; doi:10.1002/ece3.10411)

**Appendix**

Table S1. Specimens used for genetic analysis. [double-column]

X = sequenced for this study. Museum codes: LSUMZ = Louisiana State University Museum of Natural Science; NMNH = National Museum of Natural History, Smithsonian Institute; MCZ = Museum of Comparative Zoology, Harvard; MLZ = Moore Laboratory of Zoology, Occidental College; FMNH = Field Museum of Natural History, Chicago; MZFC = Museo de Zoología "Alfonso L. Herrera," Universidad Nacional Autónoma de México; QCAZ = Museo de Zoología QCAZ, Pontificia Universidad Católica del Ecuador.; UWBM = Burke Museum, University of Washington

Table S2. Principal components analysis of morphological data [single-column]


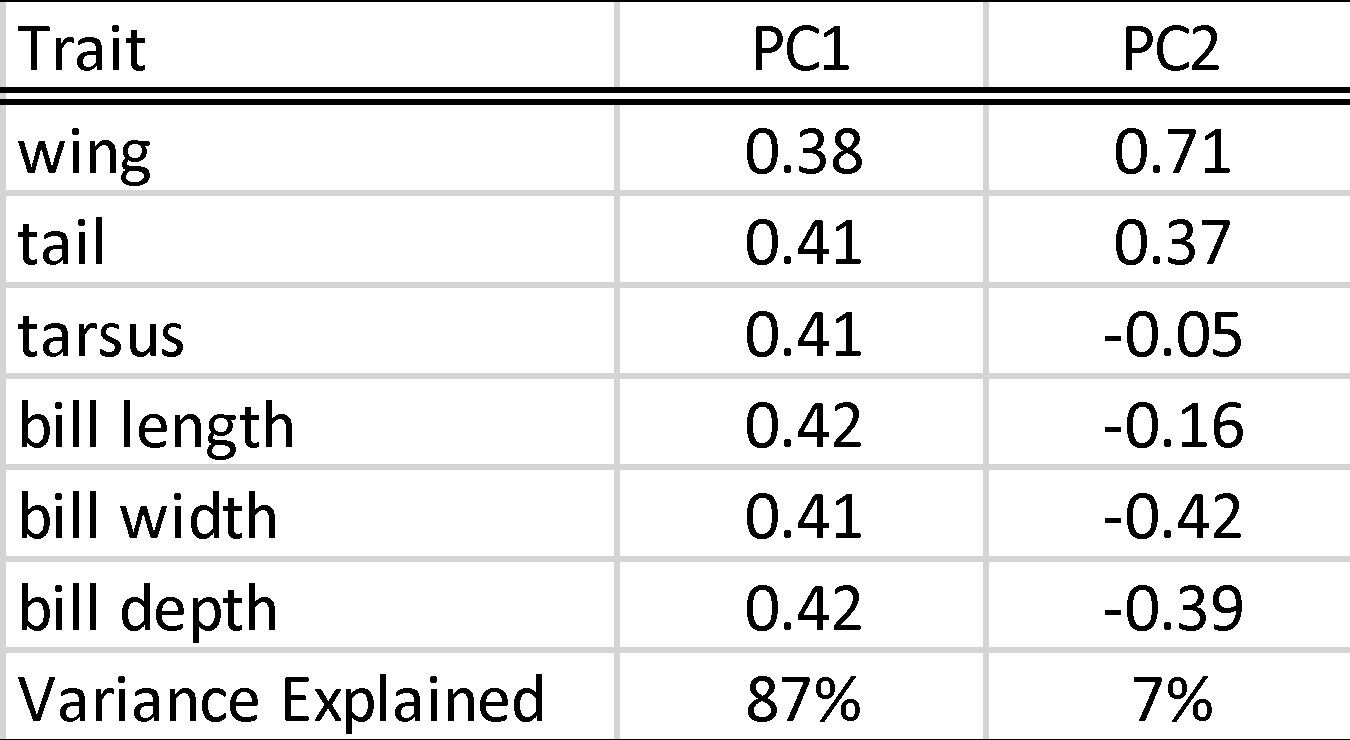


Table S3. Principal components analysis of vocalization data [single-column]


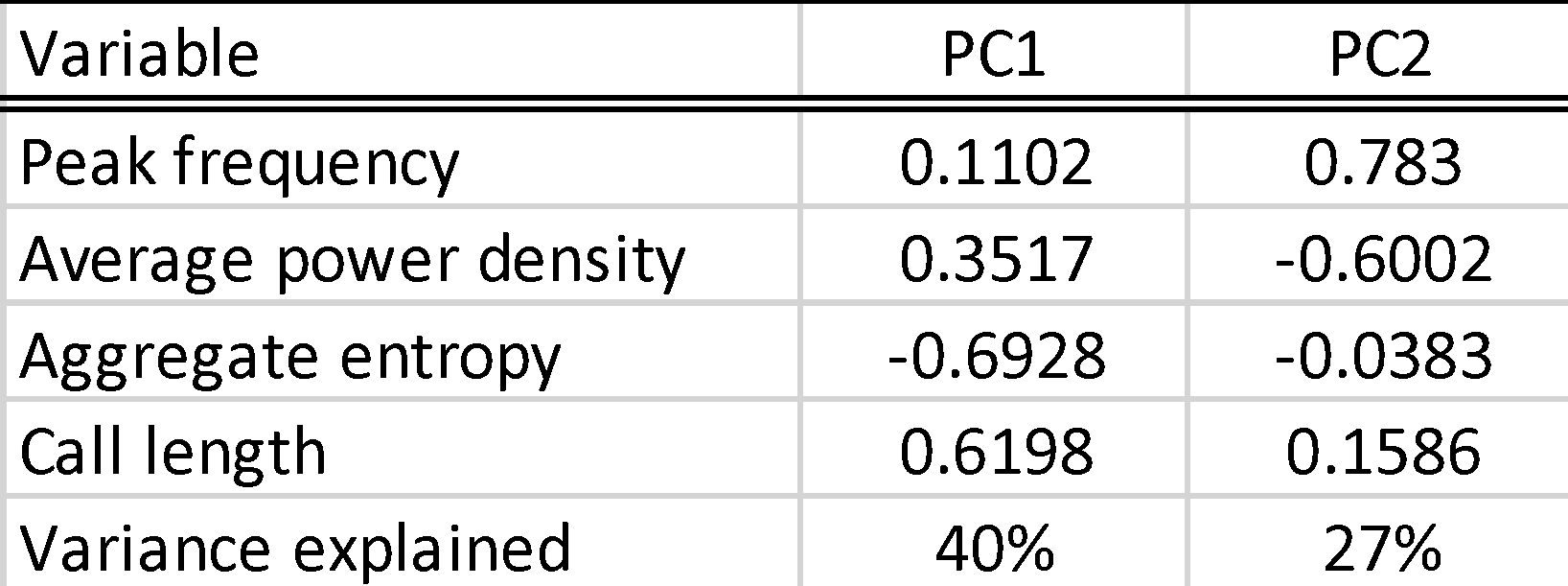


Fig. S1. PCA results for males and females showing similar patterns of geographic variation for each sex. Figure legend is the same as in Fig. 5.


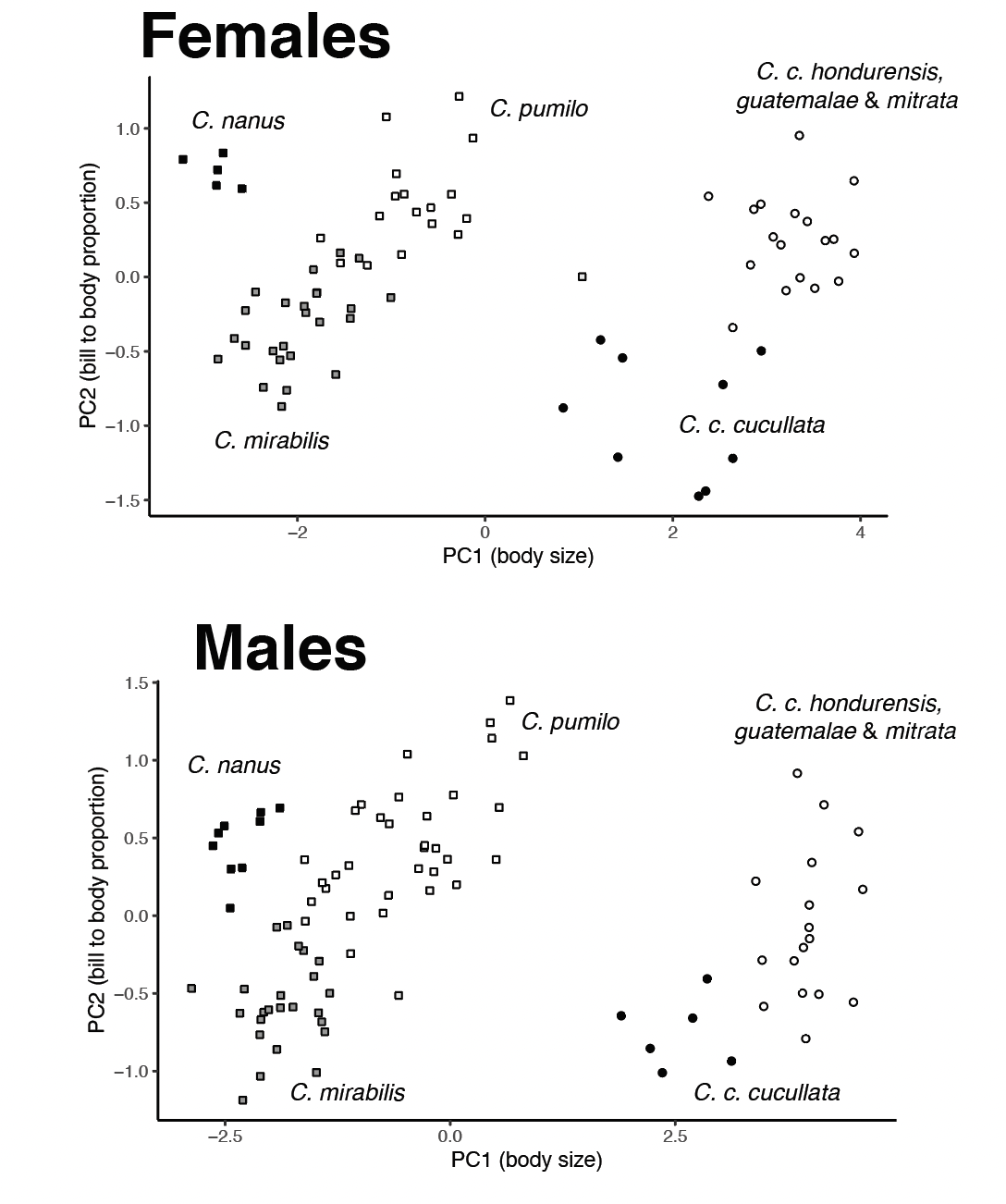


Fig. S2. Neotype specimen for *Cyanolyca mitrata*: MLZ:Bird:53628 in the Moore Laboratory of Zoology, Occidental College, Los Angeles, California. Photo by Marky Mutchler.


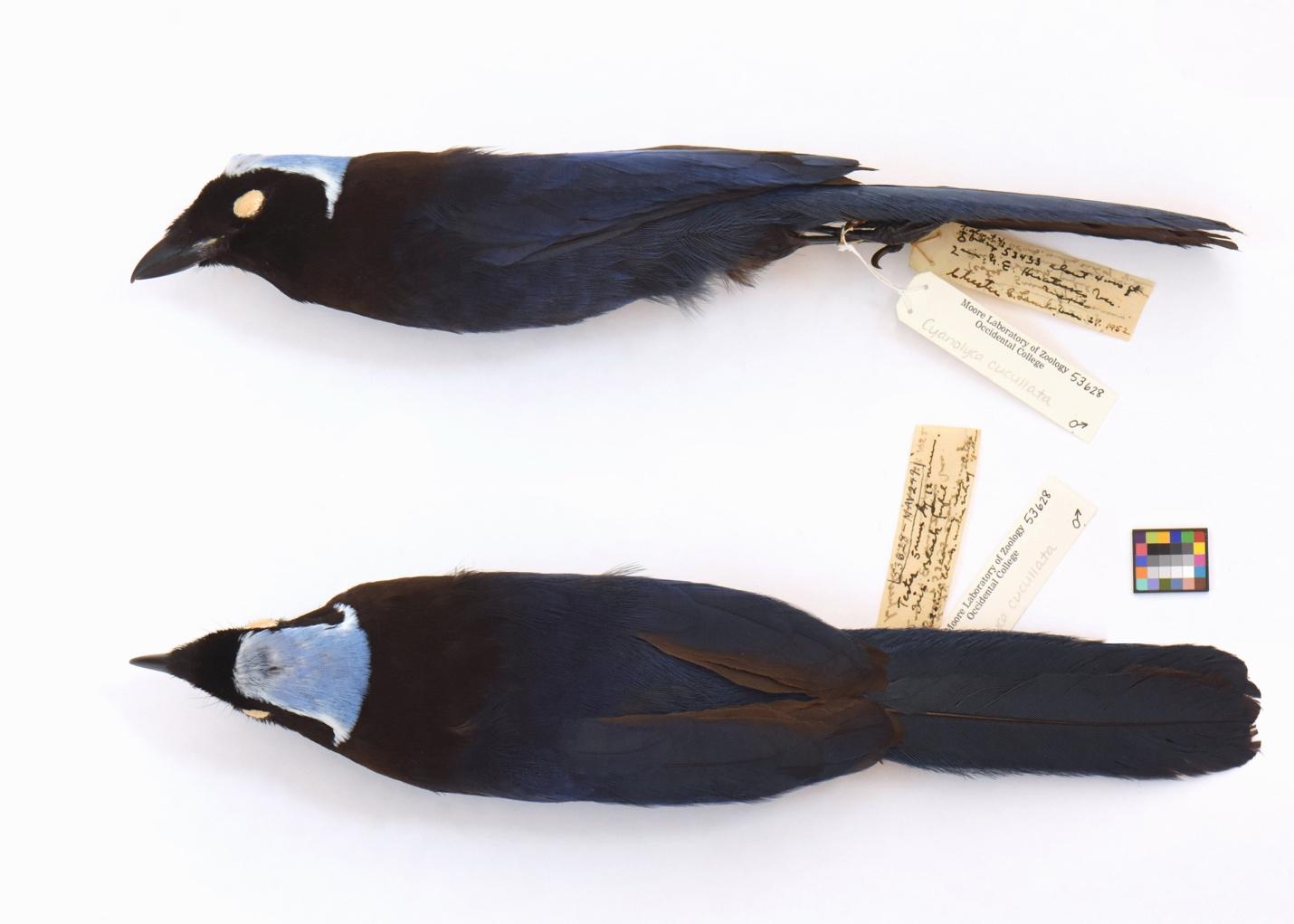

Supplement: Supplementary file 2 — Appendix S1: [file ECE3-13-e10411-s001.docx]
